# Human ribosomal P1-P2 heterodimer represents an optimal docking site for ricin A chain with a prominent role for P1 C-terminus

**DOI:** 10.1038/s41598-017-05675-5

**Published:** 2017-07-17

**Authors:** Przemysław Grela, Xiao-Ping Li, Patrycja Horbowicz, Monika Dźwierzyńska, Marek Tchórzewski, Nilgun E. Tumer

**Affiliations:** 1 Department of Plant Biology, School of Environmental and Biological Sciences, Rutgers University, New Brunswick, New Jersey, 08901-8520 USA; 2 Department of Molecular Biology, Maria Curie-Skłodowska University, Akademicka 19, 20-033 Lublin, Poland

## Abstract

The eukaryotic P-stalk contains two P1-P2 protein dimers with a conserved C- terminal domain (CTD) critical for the interaction with external factors. To understand the role of the individual CTD of human P1/P2 proteins, we examined the interaction of reconstituted human P-protein complexes and C-terminally truncated forms with ricin A chain (RTA), which binds to the stalk to depurinate the sarcin/ricin loop (SRL). The interaction between P-protein complexes and RTA was examined by surface plasmon resonance, isothermal titration calorimetry, microscale thermophoresis and bio-layer interferometry. The P1-P2 heterodimer missing a CTD on P2 was able to bind RTA. In contrast, the P1-P2 heterodimer missing the CTD of P1 protein displayed almost no binding toward RTA. Very low interaction was detected between RTA and the non-truncated P2-P2 homodimer, suggesting that the structural architecture of the P1-P2 heterodimer is critical for binding RTA. The reconstituted pentameric human stalk complex had higher affinity for RTA than the P1-P2 dimer. Deletion of P1 CTD, but not P2 CTD reduced the affinity of the pentamer for RTA. These results highlight the importance of the heterodimeric organization of P1-P2 in the human stalk pentamer and functional non-equivalence of the individual P-protein CTDs in the interaction with RTA.

## Introduction

The ribosomal stalk is a pentameric protein complex made up of universally conserved ribosomal protein uL10 and P1, P2 proteins, which form a pentameric architecture called the P-stalk, uL10-(P1-P2)_2_, located on the large subunit of eukaryotic ribosome^1–5^. In higher eukaryotes, P1 and P2 proteins are attached to the uL10 protein in the form of two heterodimers. The uL10 according to new nomenclature, the former name P0^6^, forms backbone of the stalk. Lower eukaryotes, such as *Saccharomyces cerevisiae*, possess two P1/P2 protein forms, P1A, P1B, P2A and P2B, which form two dimers, P1A/P2B and P1B/P2A, bound to two specific contiguous sites on the uL10 protein^7, 8^. The P-proteins together with ribosomal uL10 and uL11 protein and the sarcin-ricin loop (SRL) are part of the GTPase Associated Center (GAC)^9–12^. The P-stalk is considered to be the one of the main functional elements in the GAC and is thought to be responsible for the recruitment of translational GTPases (trGTPases), such as elongation factor 2 (EF2) and stimulation of factor-dependent GTP hydrolysis^11, 13–16^. The SRL is also a critical component of the GAC^17–19^ and is the key ribosomal element that activates the elongation factor G (EF-G), by placing critical His87 into its proper position for GTP hydrolysis on bacterial ribosomes^19^. A_2660_ of the SRL (*E. coli* numbering) is involved in extensive interaction network in the close vicinity of the GTP active site.

The most prominent feature of the eukaryotic stalk proteins is a conserved amino acid element present at the C-terminal domain (CTD) of uL10 and P1/P2 proteins^5^. This stretch of highly acidic and hydrophobic amino acids (EEEAKEESDDDMGFGLFD) is regarded as the main functional element of the stalk proteins and is involved in the interaction with translational GTPases and ribosome inactivating proteins (RIPs)^20^. Ricin is a type II RIP consisting of two subunits, an enzymatically active A-chain (RTA) linked by a disulfide bond to a B-chain (RTB) that facilitates uptake of the toxin by mammalian cells^21^. RTA is an RNA *N*-glycosidase that specifically removes an adenine from a universally conserved SRL in the 28 S rRNA^22–24^. RTA interacts with P-proteins of the ribosomal stalk to depurinate the SRL in yeast and in human cells^25–27^. Several functionally related RIPs, such as Shiga toxin 1 (Stx1)^26, 28^, Shiga toxin 2 (Stx2)^29, 30^, trichosanthin (TCS)^31–33^ and maize RIP^34^ interact with the ribosomal P-protein stalk to depurinate the SRL, while pokeweed antiviral protein (PAP) interacts with ribosomal protein L3 to access the SRL^35^. The deletion of P-proteins reduces the depurination activity and cellular sensitivity to RTA, indicating that binding to the P-stalk is a critical step in depurination of the SRL and in the toxicity of ricin^25, 36, 37^. The dissociation constant (*K*_D_) of RTA interaction with the yeast ribosomal stalk pentamer is in the low nanomolar range, indicating that P-proteins represent a primary landing platform for ricin to accelerate depurination of the SRL^38^.

Although, the SRL is highly conserved, the P-proteins determine the ribosome specificity of ricin and other RIPs^20, 21^. The P-proteins also determine the specificity of ribosomes towards the trGTPases. The replacement of prokaryotic stalk proteins with the eukaryotic P-protein complex changed the elongation-factor-specificity of *E. coli* ribosomes from prokaryotic EF-G to eukaryotic EF2^39, 40^ and rendered *E. coli* ribosomes, which are insensitive to TCS, susceptible to depurination by TCS^41^. Using TCS as experimental model, the interaction site of P-proteins with RIPs was mapped to a conserved 11-mer peptide, SDDDMGFGLFD (P11) present at the CTD of all P proteins^32^. This interaction is required for the full activity of TCS and is primarily mediated by the electrostatic interactions of K173, R174 and K177 in the C-terminal domain of TCS with the conserved DDD residues in the CTD of P-proteins, along with hydrophobic interactions, which play vital role in stabilization of bilateral contacts between TCS and P11^26, 33^. The crystal structure of RTA with a 6-mer peptide corresponding to the conserved last six residues of the stalk proteins (GFGLFD) showed that the peptide docks into a hydrophobic pocket at the C-terminus of RTA^42, 43^. The structural superposition of the TCS-P2 and RTA-P2 complexes demonstrated that the P2 peptide adopted distinct orientations and different interaction modes with the two different RIPs, suggesting the flexibility of the CTD in facilitating the access of RIPs to the ribosome^42, 43^.

Although, the involvement of the P-proteins and their C-termini in RIP binding has been documented, the biochemical studies were performed exclusively with isolated experimental systems using various oligomeric forms of individual P-proteins, such as homodimers of P2^32^, oligomers P1 or uL10^31^ or peptides^26, 32, 33, 42, 43^. The P-stalk proteins preferentially form biologically relevant P1-P2 heterodimers, which can be regarded as the central functional element of the ribosomal stalk^44–47^. Moreover, the P1-P2 heterodimers are attached to the ribosome via the uL10 protein, and the final structural organization of P1-P2 is formed after binding to uL10^7^. The interaction of RIPs with structural variants of the human P1-P2 dimer or with the uL10(P1-P2)_2_ pentamer has not been previously examined. Here we dissected the interaction of RTA with the human P1-P2 dimer and with the reconstituted human stalk pentamer and their truncated forms missing the CTD of the individual P1 and P2 proteins. We show that the heterodimeric conformation of P1-P2 in the stalk pentamer represents an optimal binding site for RTA and present the first evidence that universally conserved CTD of P1 and P2 have an unequal role in their interaction with RTA.

## Results

### Human P1-P2 complexes do not respond equally to deletion of their conserved C-terminal fragment in their interaction with RTA

**Figure 1:**
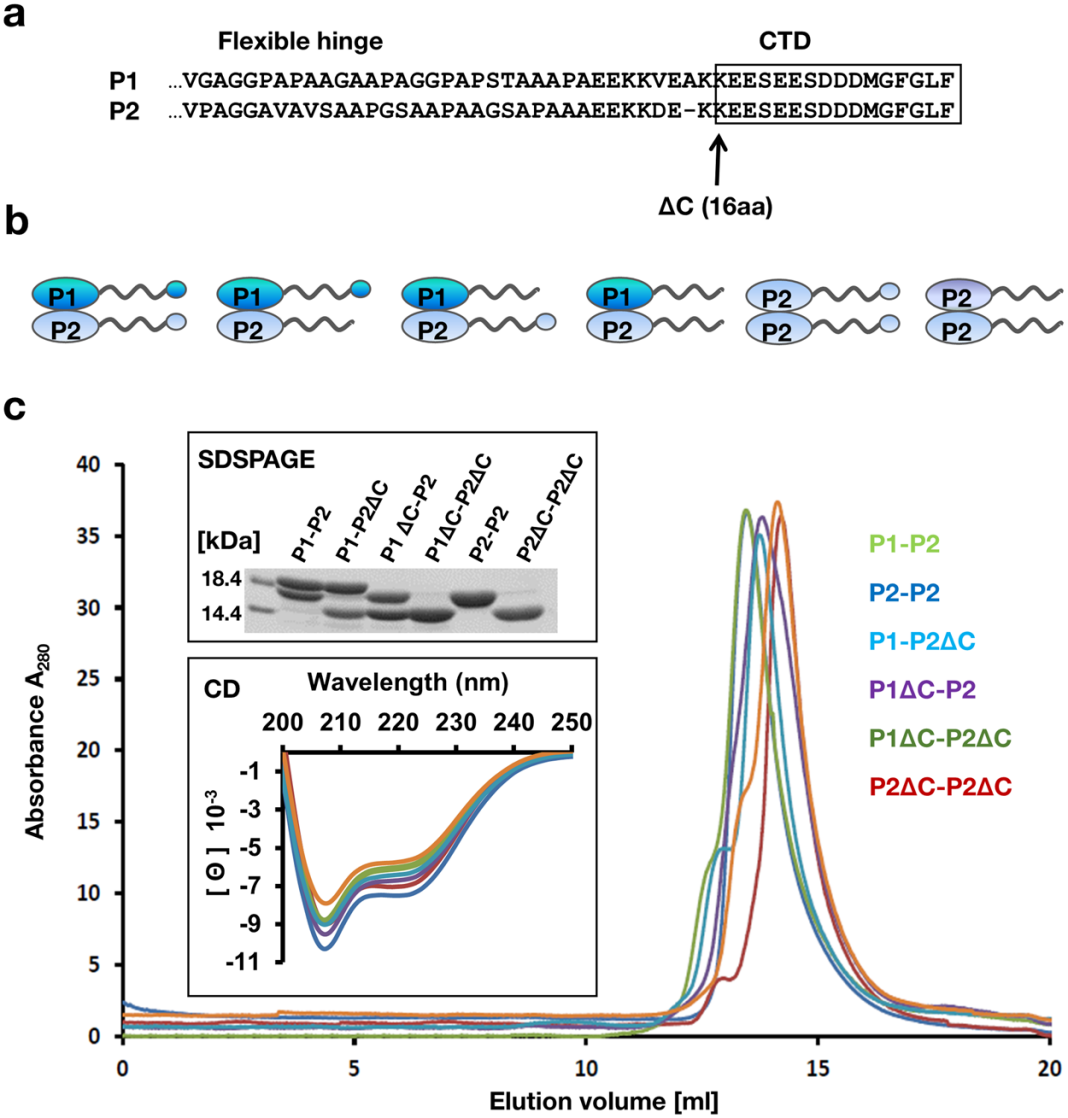
Characterization of human ribosomal stalk P1-P2 complexes. (**a**) Alignment of C-terminal amino acid sequences of human ribosomal P1, P2 protein. The arrow indicates the deletion site for the last 16 amino-acids from the C-termini (ΔC). (**b**) Models of human ribosomal P1-P2 and P2-P2 complexes deleted in the conserved C-terminal sequences (16aa). **(c)** Size exclusion chromatography (SEC) of recombinant human P1-P2 protein complexes. Purified complexes (10 μg of protein) were analyzed by SDSPAGE (upper panel) and circular dichroism (CD) (lower panel). The full-length image of the cropped SDSPAGE gel is shown in Fig. S6.

Sequence alignment of P1 and P2 proteins shows a conserved C-terminal region rich in acidic and hydrophobic residues, which was shown to be involved in the interactions with the translational GTPases (trGTPases)^5^ and the RIPs^21^ (Fig. 1a). To evaluate the interaction between human P1/P2 proteins and RTA we constructed heterodimers of human P-proteins, which are regarded as the smallest functional unit of the stalk structure. Additionally, a set of structural variants of P1 and P2 proteins lacking the conserved 16-amino acids at the C-terminus were prepared (Fig. 1b). The individual P-proteins were expressed in *E. coli*, purified and assembled to obtain heterodimers^44, 45, 48^. All complexes were examined for their purity and structural parameters using SDS-PAGE, size exclusion chromatography (SEC), circular dichroism (CD) (Fig. 1c), and native-mass spectrometry (native-MS) (Table 1). The following complexes were used: the intact P1-P2 heterodimer and truncated forms, P1ΔC-P2, which is lacking 16-amino acids on the C-terminus of the P1, P1-P2ΔC, which is lacking 16-amino acids from the C-terminus of P2, and P1ΔC-P2ΔC, where all conserved CTDs were removed from P1 and P2. We also used P2 protein, since P2 spontaneously forms a homodimer in the absence of its P1 partner^44^, and its truncated form, P2ΔC-P2ΔC. All complexes were purified to homogeneity and displayed monodispersity, as shown by SEC analysis (Fig. 1c). Far-UV spectra by CD analysis showed that all complexes maintained structural properties, resembling those for the full-length intact complex^44^. Finally, we have applied native-MS approach to verify the molecular masses of the protein complexes within single Dalton resolution. We used mild ionization conditions, which allowed us to observe intact protein-complexes in the gas-phase. The obtained molecular masses of all complexes agreed with the calculated ones (Table 1) and showed that the recombinant proteins are in intact form, with one modification most likely indicating posttranslational removal of the first methionine^44, 49^.

**Table 1:** Molecular masses of protein species detected by native mass spectrometry.

Protein complex	MMcal (Da)^*^	MMmeasured (Da)*
P1-met	12010.6	12009.98 ± 06
P1ΔC-met	10357.8	10357.52 ± 0.57
P2	12292.7	12292.66 ± 0.10
P2ΔC-met	10357.8	10357.68 ± 0.10
P1-met-P2	24302.6	24302.45 ± 7.14
P1-met-P2ΔC-met	22368.4	22367.97 ± 2.54
P1ΔC-met-P2	22499.5	22497.71 ± 5.11
P1ΔC-met-P2ΔC-met	20564.6	20563.95 ± 11.51
P2-P2	24585.4	24586.09 ± 8.81
P2ΔC-met-P2ΔC-met	20715.7	20727.95 ± 11.10

*Calculated molecular masses (MMcal) compared with molecular masses

determined by native-MS (MMmeasured).

RTA was expressed in *E. coli* as an N-terminal 10xHis-tagged fusion. Sample homogeneity was confirmed by SDS-PAGE and monodispersity by SEC (Fig. S1). The structural parameters were verified by CD, which showed that RTA displays well folded β-structure, as determined by crystallography (Fig. S1, CD inset)^50, 51^. The native-MS showed that the measured molecular mass of RTA (31337.85 ± 0.60 Da) was in good agreement with the calculated one (31412.4 Da), indicating possible removal of the first methionine from the recombinant protein.

**Figure 2:**
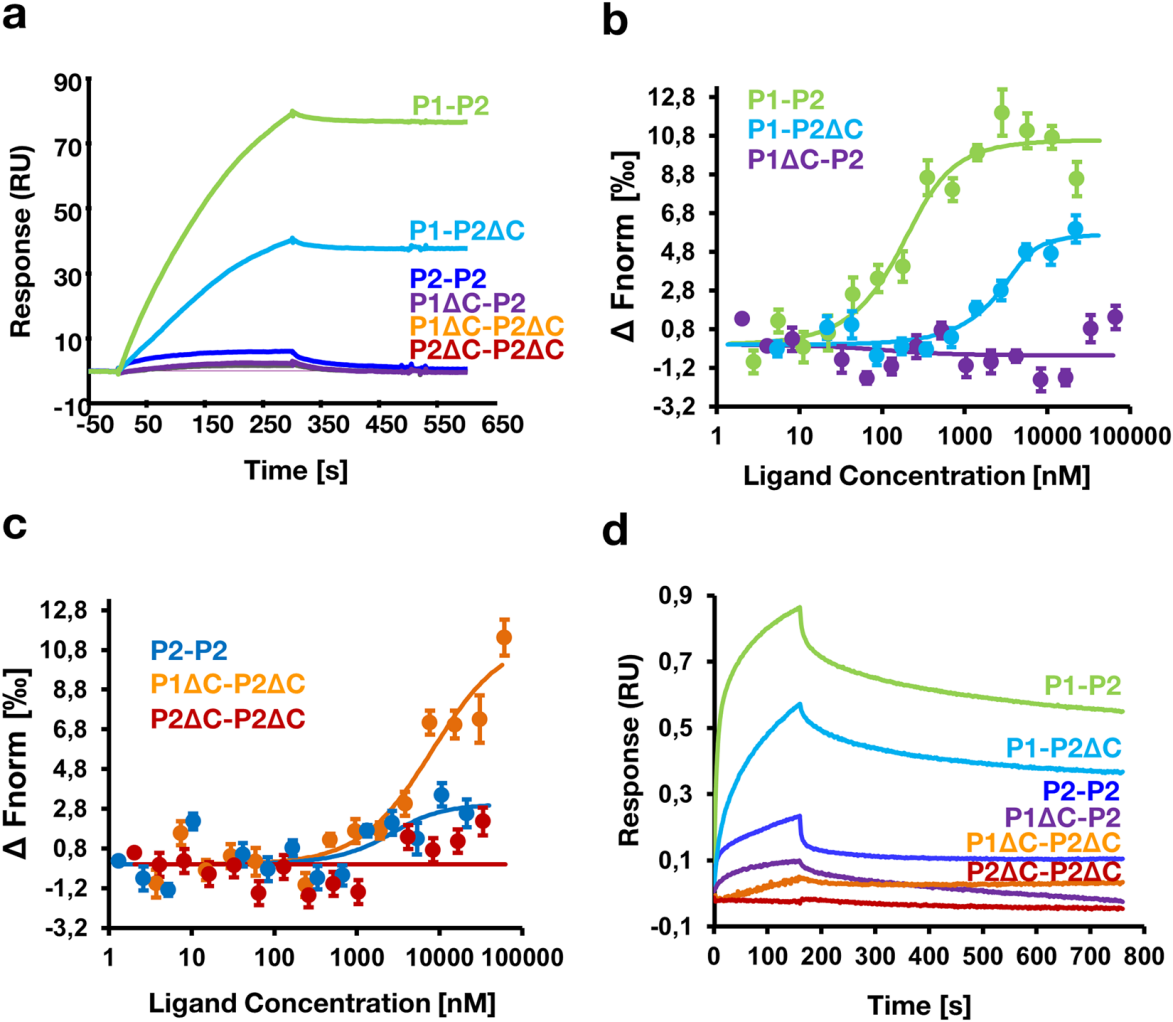
Interaction of RTA with human ribosomal stalk P1-P2 complexes. (**a**) Interaction of RTA with different forms of human P1-P2 dimers measured with surface plasmon resonance (SPR). Interaction was analyzed using Biacore T200. The His-tagged RTA was immobilized on the Ni^2+^–nitrilotriacetic acid (NTA) sensor chip as the ligand and the human P1-P2 complexes were used as an analyte. P1-P2 dimers (50 nM) were passed over the immobilized RTA at 50 µL/min for 5 min and dissociation was measured for 3 min. (**b**) Microscale thermophoresis (MST) binding curves for the P1-P2, P1-P2ΔC and P1ΔC-P2 complexes with RTA. **(c)** MST binding curves for the P2-P2, P2ΔC-P2ΔC and P1ΔC-P2ΔC and RTA. *ΔFnorm* values were centered for every curve at 0. Error bars ± SE (*n* = 3). (**d**) Interaction of RTA with human ribosomal stalk P1-P2 complexes measured with bio-layer interferometry (BLI). Interaction was analyzed using OCTET 96 RED with NTA chip sensors. Representative association/dissociation curves are shown for the dimeric complexes at 112 nM. Data are plotted as means of three experiments.

To examine the role of the CTD of P-proteins on the interaction with RTA, we used surface plasmon resonance (SPR) with Biacore T200. RTA was captured on NTA chip as ligand and P-protein complexes were used in the mobile phase as the analyte (Fig. 2a). The analysis showed that intact human P1-P2 heterodimer interacts with RTA with fast association, but without apparent fast dissociation. The lack of 16 amino acids from the CTD of the P2 protein (P1-P2ΔC) diminished binding of RTA, but lack of CTD of P1 protein (P1ΔC-P2) almost completely eliminated the interaction (Fig. 2a). The binding profile of P1 and P2 lacking CTD of both proteins (P1ΔC-P2ΔC) was superimposable on that of P1ΔC-P2. RTA showed very low interaction with the P2-P2 homodimer (Fig. 2a). An even lower level of interaction was observed P2ΔC-P2ΔC, which contained a deletion of both CTDs. These results showed unequal role of the conserved CTD of P1 and P2 in binding RTA. Binding was reduced when CTD of P2 was deleted, but was almost completely abolished when CTD of P1 is deleted.

To confirm the Biacore data, we used isothermal calorimetry (ITC) to investigate the interaction between RTA and the P-protein complexes in solution. Since RTA has a tendency to aggregate at high protein concentration, we used a low concentration of RTA in the cell and titrated human protein complexes in 1:10 ratio to RTA. Only in the case of experimental set-up where P1-P2 heterodimer was titrated onto RTA in the cell, we observed an efficient exothermic process, as indicated by the raw ITC data. The dilution-corrected and integrated heat data were best-fitted with a one-site binding model, as shown in the nonlinear regression fit (Fig. S2). The analysis confirmed that P1-P2 dimer represents a structure capable of efficiently interacting with RTA with 1:1 stoichiometry. A low level of interaction was observed when P2 protein lacked its C-terminal tail (P1-P2ΔC), but not when P1 protein lacked its C-terminal tail, confirming a pivotal role of P1 protein in the interaction with RTA. For the rest of truncated variants of P1-P2 as well as P2-P2 complexes, no binding was detected using equivalent concentrations of the components.

We further examined the interaction using microscale thermophoresis (MST), which allowed us to work within pM rage of protein concentration due to its high sensitivity. RTA was labeled with NT-647 fluorescent dye and titrated with the different P-protein complexes. We obtained a fully saturated curve with an apparent dissociation constant (*K*_D_) of 86 nM with the P1-P2 heterodimer (Fig. 2b). Using the same experimental setup, we were able to obtain good fit of the experimental data for the P1-P2ΔC complex, which showed *K*_D_ about 6 times higher at 533 nM (Fig. 2b). P2-P2 homodimers displayed low, but still specific interaction with RTA having *K*_D_ in a range of 1–2 µM (Fig. 2c). Binding was not observed with the double deletion mutant, P1ΔC-P2ΔC, at low concentrations and saturation was not reached at the higher concentrations. Thus, it was not possible to fit the experimental data and calculate the dissociation constant. RTA did not show interaction with the P1ΔC-P2 or the P2ΔC-P2ΔC complex.

To validate the kinetic parameters, we examined the binding of RTA to P1-P2 dimers by bio-layer interferometry (BLI). RTA was captured on NTA sensor chip as ligand and P1-P2 protein complexes were used as the analyte. Binding curves for a concentration series of P1-P2 dimers and the corresponding fits to the 1:1 interaction model are shown in Fig. S3. Consistent with previous results, BLI clearly confirmed highest specificity for RTA toward the intact P1-P2 dimer, with an apparent *K*_D_ of 21 nM (Fig. 2d and Table 2). The binding amplitude (expressed in nanometers) obtained for the P1-P2ΔC complex was lower, but still displayed a *K*_D_ of 90 nM (Fig. 2d and Table 2). Similar to previous kinetic analysis, P2-P2 dimer showed very low, but detectable interaction in range of *K*_D_ = 2 µM. Almost no binding of RTA was observed with the P1ΔC-P2 complex or with the double deletion mutants, P1ΔC-P2ΔC and P2ΔC-P2ΔC complexes. Deletion of the last 16 amino acids from the CTD of the P2 protein reduced the association rate constant (*k*_on_) of the interaction with P1-P2ΔC (*k*_on_ = 3.54 ± 0.04 × 10^4^) compared to the intact P1-P2 (*k*_on_ = 1.75 ± 0.39 × 10^5^) (Table 2). The effect was more prominent in RTA vs P2-P2 homodimer interaction where *k*_on_ and *k*_off_ were reduced about an order of magnitude (*k*_on_ = 1.72 ± 0.09 × 10^4^, *k*_off_ = 3.85 ± 1.87 × 10^−2^) compared to the intact P1-P2 complex (*k*_on_ = 1.75 ± 0.39 × 10^5^, *k*_off_ = 3.71 ± 0.45 × 10^−3^) (Table 2). The obtained data unequivocally show that P1-P2 dimer represents a favorable structural form recognized by RTA with a binding affinity in the nanomolar range, with significant contribution of the CTD of P1 protein.

**Table 2:** Kinetic parameters of the interaction of RTA with human stalk complexes analyzed by bio-layer Interferometry.

Protein complex	*K_D_ (M^−1^)	*k_on_ (M^−1^ s^−1^)	*k_off_ (s^−1^)
P1-P2	(2.18 ± 0.24) × 10^−8^	(1.75 ± 0.39) × 10^5^	(3.71 ± 0.45) × 10^−3^
P1-P2ΔC	(9.03 ± 0.17) × 10^−8^	(3.54 ± 0.04) × 10^4^	(3.19 ± 0.04) × 10^−3^
P1ΔC-P2	N/A	N/A	N/A
P1ΔC-P2ΔC	N/A	N/A	N/A
P2-P2	(2.19 ± 0.97) × 10^−6^	(1.72 ± 0.09) × 10^4^	(3.85 ± 1.87) × 10^−2^
P2ΔC-P2ΔC	N/A	N/A	N/A
ΔuL10(P1-P2)_2_	(4.07 ± 1.99) × 10^−9^	(4.19 ± 1.98) × 10^5^	(1.31 ± 0.03) × 10^−3^
ΔuL10(P1-P2ΔC)_2_	(5.32 ± 0.03) × 10^−9^	(2.29 ± 0.01) × 10^5^	(1.22 ± 0.05) × 10^−3^
ΔuL10(P1ΔC-P2)_2_	(1.12 ± 0.02) × 10^−8^	(1.94 ± 0.27) × 10^5^	(2.16 ± 0.26) × 10^−3^

*K_D_, *k*_on_ and *k*_off_ values represent the average of three replicate experiments.

### Pentameric organization of human uL10(P1-P2)_2_ complex represents optimal conformation for the interaction with RTA with significant contribution of the CTD of P1 protein

**Figure 3:**
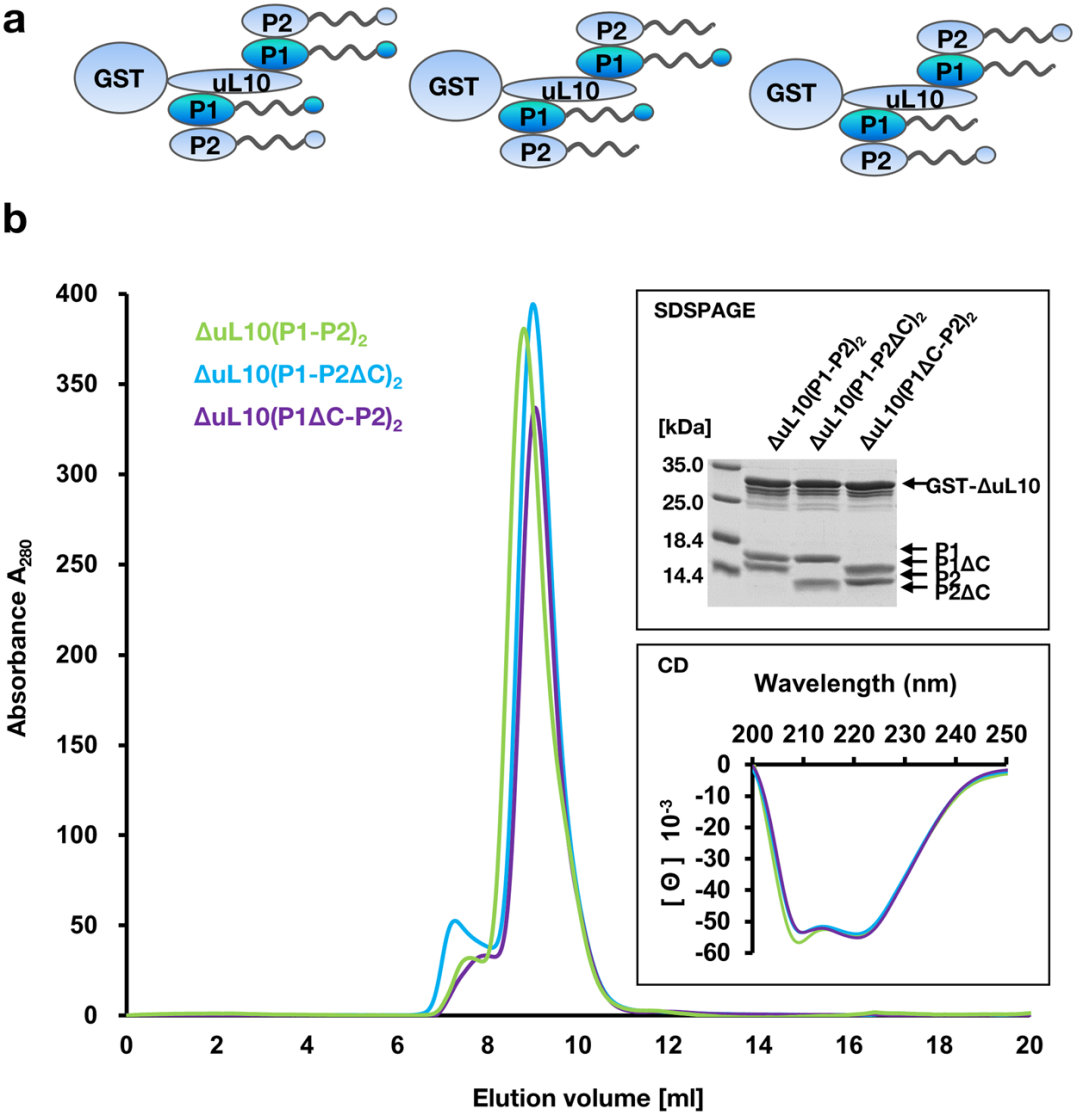
Characterization of human ribosomal stalk pentamer ΔuL10(P1-P2)_2_ complexes. **(a)** Models of human ribosomal stalk pentamer complexes deleted in conserved C-terminal sequences (16aa) of P1 or P2. **(b)** Size exclusion chromatography (SEC) of recombinant stalk pentamer ΔuL10(P1-P2)_2_ complexes. Purified complexes (10 μg of protein) were subjected to SDSPAGE (upper panel) and circular dichroism (CD) (lower panel). The full-length image of the cropped SDSPAGE gel is shown in Fig. S7.

To evaluate the binding of the pentameric stalk complex with RTA we have assembled the human pentameric complex *in vivo* in *E. coli* using a previously developed poly-cistronic expression system^52^, where three genes for the truncated ΔuL10 and P1/P2 proteins were placed in one row for simultaneous expression of all stalk elements. The uL10/P1/P2 gene array was expressed in *E. coli* cells with ΔuL10 lacking the rRNA binding domain as hybrid proteins with an N-terminal GST-tag. The expression system allowed expression of the full-length P1/P2 proteins, as well as structural variants of P1 and P2 proteins lacking the conserved 16-amino acids at the CTD (Fig. 3a). The following complexes were obtained: the pentameric ΔuL10(P1-P2)_2_ complex and truncated forms: ΔuL10(P1ΔC-P2)_2_, which is lacking 16-amino acids from the CTD of P1and ΔuL10(P1-P2ΔC)_2_, which is lacking 16-amino acids from the CTD of P2. All complexes were purified to homogeneity and displayed monodispersity, as shown by SEC analysis (Fig. 3b). The calculated molecular masses using analytical SEC indicated that the complexes have pentameric organization (Fig. 3b). The complexes were examined for their purity and structural parameters using SDS-PAGE and CD (Fig. 3b). The CD spectra of all protein complexes displayed spectra with double minima at 208 and 222 nm (Fig. 3b), indicating structural coherence between all pentameric variants. All protein complex elements were identified within single Dalton resolution using the MS approach. However, the pentameric complex was unstable in gas-phase, and its intact mass was not measured.

**Figure 4:**
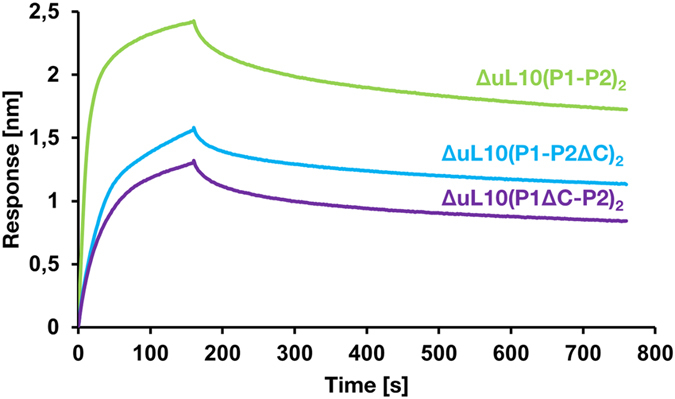
Interaction of RTA with the human ribosomal stalk pentamer complexes. Interaction of RTA with different forms of human stalk pentamer ΔuL10(P1-P2)_2_, ΔuL10(P1ΔC-P2)_2_ and ΔuL10(P1-P2ΔC)_2_ was analyzed by BLI using the OCTET 96 RED system. The His tagged RTA was immobilized on an Ni^2+^-nitrilotriacetic acid (NTA) sensor chip as the ligand and human stalk pentamers were used as the analyte. Representative association/dissociation curves are shown for the stalk complexes at 112 nM. The curves represent the means of three experiments and were obtained by simultaneously fitting the association and dissociation responses to a 1:1 binding model.

To examine the interaction of the various forms of pentameric complexes with RTA, BLI was conducted to determine the binding affinity parameters. RTA was captured on an NTA chip as the ligand and P-protein complexes with deleted CTD were used as the analyte. Binding curves for a concentration series of human stalk pentamer and C-terminally deleted forms are shown in Fig. S4. The results showed that the intact human ΔuL10(P1-P2)_2_ complex interacts with RTA with a fast association and a fast dissociation profile with an apparent dissociation constant of *K*_D_ = 4 nM (Fig. 4 and Table 2). The lack of 16 amino acids from the CTD of the P2 protein in the ΔuL10(P1-P2ΔC)_2_ complex diminished binding of RTA with a *K*_D_ of 5 nM. The lack of CTD of P1 protein in the ΔuL10(P1ΔC-P2)_2_ complex exerted the strongest impact on *K*_D_, lowering it to 11 nM (Fig. 4, Table 2). The association (*k*_on_) rate constant was reduced to half when CTD of P2 was deleted in ΔuL10(P1-P2ΔC)_2_, while the dissociation rate (*k*_off_) was not affected. In contrast, *k*_on_ was reduced to half and *k*_off_ was increased 2-fold when the CTD of P1 was deleted in ΔuL10(P1ΔC-P2)_2_. These data indicate that the intact pentameric complex and ΔuL10(P1-P2ΔC)_2_ variant display more similar binding kinetics compared to the ΔuL10(P1ΔC-P2)_2_ variant, which exhibits slower *k*_on_, faster *k*_off_ and reduced affinity for RTA (Table 2). The binding parameters of the pentameric complexes showed that the pentameric architecture of the stalk complex represents an optimal structural arrangement to interact with RTA and confirmed the prominent role of P1 CTD as the key element in the interaction with RTA.

## Discussion

The ribosomal P-proteins are the main docking platform for a very broad family of translational factors conferring full functionality for the ribosome. The P-proteins are also targets for several RIPs to gain access to the ribosome and to depurinate the SRL. Previous studies examined the interaction of RIPs with individual P-proteins^31, 32, 53^ or short peptides^26, 33, 42, 43^. The interaction of RIPs with the intact P1-P2 dimer has not been previously examined. Moreover, the role of individual CTDs of P-proteins in native heterodimer conformation has not been investigated. Shiga-like toxin 1 (Stx1) was shown to interact with individual forms of uL10, P1 and P2 proteins, especially with an 11-mer peptide (P-11) corresponding to the conserved CTD of the ribosomal P-stalk proteins^26^. However, since the optimal structural configuration of the P-stalk proteins is exhibited by heterodimeric and especially pentameric organization^4, 38, 44, 49^, we examined here the interaction of RTA with the human P1-P2 heterodimer, which is regarded as the smallest functional component of the eukaryotic stalk. We also examined the interaction of RTA with the human pentameric stalk complex, where P1-P2 dimers are attached to the uL10 protein. Our results show that the human P1-P2 dimer represents a primary docking site for RTA, with a dissociation constant in the nanomolar range. The ITC analysis showed that one molecule of RTA interacts with a single P1-P2 dimer, suggesting that only one CTD is sufficient for the interaction with RTA. These results are consistent with previous studies, which showed that RTA interacts with yeast ribosomes via fast and slow interactions, where the fast interactions occur with the stalk P-proteins^54^.

The human P1/P2 stalk proteins represent a class of partially disordered proteins, where well-structured α-helical NTD responsible for their dimerization is connected to a very flexible, unstructured C-terminal tail with highly conserved residues (SDDDMGFGLFD)^5, 55^. Such disordered domains are believed to play important roles in protein function by virtue of their ability to adapt themselves to the requirements of different binding partners by applying an induced fit mechanism, thereby conferring high specificity to the interaction. It has been argued that the existence of multiple copies of the conserved CTD of the stalk structure, which protrude outward from the ribosomes to the cytoplasm, functions to fetch the external factors and draw them into the ribosome^14, 16, 41^. The molecular mechanism of the interaction still remains unknown as well as the reason for multiplication of the CTD in all P-proteins. The multiplicity raises the question whether all CTD of the stalk proteins are equal in function. Several studies have addressed this issue, showing that P1 protein is internally located with restricted reactivity while P2 protein is more external and accessible to interact with other cellular components^41, 55, 56^. Additionally, it was shown that P1 and P2 differently contribute to ribosomal activity dependent on eEF-1a and eEF-2^57^. We compared the interaction of RTA with the yeast pentameric complex containing two pairs of P1/P2 or the trimeric stalk complexes containing only one pair and showed that pentameric organization accelerates recruitment of RTA to the ribosome^38^. However, our understanding of P1/P2 proteins *modus operandi* is still far from complete. Here, we examined the role of the individual CTD of P1 and P2 proteins within the native heterodimeric and pentameric conformations in the interaction with RTA. Analysis of truncated P1-P2 dimers showed that the CTD of P1 protein is more critical than the CTD of P2 protein for efficient interaction of the stalk complex with RTA.

The differential binding capacity of the P1 and P2 CTD of the P1-P2 dimer to RTA could be explained by the unique behavior of the disordered nature of the CTD of P1 and P2 proteins, especially the hinge region. As shown for the analogous bacterial bL12 proteins, the (bL12)_2_ dimer is composed of the two NTD and CTD domains connected by two flexible hinges rich in glycine/alanine amino acids, as in the eukaryotic P1-P2 dimer. The bL12 hinge has propensity to form an alpha-helix, and NMR and SAXS analyses showed that one hinge can adopt an alpha-helical structure, while the second one is in extended unstructured form^58, 59^. Thus, the two CTD of the bacterial dimer behave differently in terms of structural organization, where one CTD is free and extended and second one is restricted in its motion. Since the hinge region of the eukaryotic P1 and P2 proteins closely resembles the one found in bL12, we propose that observed differences in RTA binding to the different forms of P1-P2 and P2-P2 complexes are connected to restricted freedom of the individual CTDs, allowing only for temporary stable interaction with one P1 CTD.

The P2-P2 homodimer having two identical C-termini as in P1-P2 complex displayed low binding capacity with different binding kinetics, indicating that structural organization of the heterodimer is important for RTA binding with P1 CTD as a main docking element. The interaction of P2-P2 with RTA did not show as good fit to the 1:1 interaction model as the P1-P2 heterodimer (Fig. S3), suggesting a different mechanism for RTA binding. The differences in the interaction of the P1-P2 heterodimer and P2-P2 homodimer with RTA must lie in the subtle but essential structural differences between these dimers. Previous studies showed that P2 forms a homodimer in solution, while in the absence of P2, P1 forms high-mass oligomeric aggregates. The formation of P1-P2 heterodimer is a favorable spontaneous process in which the less stable P2 homodimer is displaced by P1 to form a more stable P1-P2 heterodimer^44, 55^. Low resolution structural SAXS analyses indicated that the human P2-P2 homodimer and P1-P2 heterodimer are structurally related to each other^60^. However, NMR structural data showed that the mode of dimerization by the NTD of the two proteins is different, showing clear asymmetry with difference in the orientation of the helix-4 in the heterodimer, which may impose different CTD orientation, and at the same time expose P1-CTD^55, 61^. Only P1-P2 dimer has the capacity for binding to uL10 to assemble the functional pentameric stalk complex^55, 61, 62^. In contrast, P2-P2 homodimer cannot interact with uL10 mainly due to lack of three exposed polar residues on helix-3 in its NTD, which are substituted by conserved hydrophobic residues in the P1 protein^55, 61^. Therefore, the P2-P2 homodimer may represent a non-functional and irrelevant complex from the translational point of view. However, it is postulated that this homodimer might have an undefined non-ribosomal role. In parasite *Plasmodium sp*. P2-P2 homo-oligomeric complex was found on the surface of infected human red blood cells and its presence is connected to stage of the parasite development in red blood cells^63^. RTA showed little binding to the P2-P2 homodimer and had very low affinity, indicating that binding to P2-P2 homodimer may not be biologically significant. These results suggest that not only the CTD, but also the spatial organization of the heterodimer allows proper RTA attachment to the complex.

The pentameric stalk complex displayed 5-fold lower *K*_*D*_ value (4 nM), compared to the dimer (20 nM), indicating that the oligomeric organization of the stalk confers full functionality to the P-proteins. The pentamer complex had 2.4-fold higher association rate compared to the P1-P2 dimer (Table 2). The faster association rate of the pentamer compared to the P1-P2 dimer may be due to four copies of the CTD on the stalk complex. Deletion of the P2 CTD in the human stalk pentamer did not affect the affinity (*K*_D_) of the truncated pentamer for RTA, compared to the native stalk pentamer, while deletion of P1 CTD reduced the affinity almost 4-fold (Table 2). Deletion of either P1 or P2 CTD reduced the association rate (*k*_on_) of the pentamer with RTA by about 2-fold compared to the native pentamer, indicating that either CTD accelerates the recruitment of RTA to the ribosome for depurination. These results are consistent with previous results with the yeast stalk pentamer and indicate that multiple copies increase the scavenging ability of the CTD for RTA^38^. However, while deletion of the P2 CTD had a slight effect on the dissociation rate (*k*_off_), deletion of the P1 CTD increased the dissociation rate by 2-fold compared to the native pentamer or the pentamer missing the P2 CTD. These results suggest that the two CTDs on the human pentamer do not bind to RTA equally. RTA may interact better with P1 CTD in the stalk pentamer because this CTD may be more accessible. Therefore, the pentamer is the optimum protein configuration and the P1 and P2 CTDs are not equal in their interaction with RTA.

Our knowledge of protein-protein interactions is largely derived from stable complexes that interact very tightly. Much less is known about short-lived interactions, especially formation of transient complexes on the ribosome^64^. The interactions between RTA and P-proteins are highly specific, however short-lived^38^. The transient nature of RTA-stalk interaction is supported by the fact that the electrostatic interactions were shown to be critical for ribosome interaction with ribotoxins^65^ and for the enzymatic activity of RIPs^66, 67^. The affinity of RTA for human P1-P2 dimer (*K*_*D*_ = 21 nM and 86 nM) and for human pentamer (*K*_*D*_ = 4 nM) was three orders of magnitude lower than its affinity for the synthetic C-terminal peptides *(K*_*D*_ = 13 µM^26^ and *K*_*D*_ = 3.4 µM and 2.3 µM for C9 and C11, respectively^42^), strongly suggesting the presence of additional stabilization elements within the heterodimer and pentamer structures. Previous results in yeast showed that the P1A-P2B and P1B-P2A dimers do not interact equally with RTA *in vivo* ^68^, suggesting that the two heterodimers may have a different architecture and their CTDs may not be accessible to external factors equally. Thus, we propose an allosteric mechanism of RTA interaction with the human stalk proteins, where the conserved CTD of P1 protein within the heterodimer is responsible for primary interaction with RTA while the second CTD of P2 might be less accessible due to possible presence of alpha helix within the hinge. Additionally, the unique structural features of NTD of human P1-P2 dimer may contribute to the stabilization of the RTA interaction, assuring efficient RTA-dimer interaction. We postulate that there is cooperation between the N-terminal and C-terminal domains of the heterodimer to bind and to stabilize the RTA molecule, and the pentameric organization of the stalk represents optimal structural architecture ensuring efficient RTA binding. Our analyses provide for the first time a mechanistic model of RTA interaction with its ribosomal partners and show that delivery of RTA to the SRL does not rely solely on the interaction with the CTD of P-proteins, but also requires an orchestrated network of interactions with the ribosomal stalk structure.

## Methods

### Protein Expression and Purification

Expression, purification of recombinant human P1, P2 proteins, truncated forms and preparation of the complexes were performed according to the procedure established previously^44^. Briefly, the proteins were expressed in *Escherichia coli* BL21(DE3)RIL cells and purified using a procedure described previously for the yeast P proteins^48^. The P1-P2 heterocomplex was prepared following the denaturation/renaturation procedure established for the yeast P protein complex^45, 47^. The human pentameric complex was prepared based on a genetic system employing a tricistronic expression cassette, where three genes for uL10, P1 and P2 proteins were placed under the control of common T7 promoter using the pGEX4T1 vector. The uL10 protein, which lacks the rRNA binding domain, was expressed as a GST-tagged fusion protein. The uL10, which lacks the last 16 amino acids from the C-termini of the P-domain (uL10_199-258_) and is able to bind the P1-P2 dimers, was used (ΔuL10). The ΔuL10(P1-P2)_2_ complex was co-expressed and self-assembled *in vivo* in *E. coli* cells. The complex was purified using affinity chromatography using GST trap (GE Healthcare) column.

RTA was expressed in *Escherichia coli* BL21(DE3)RIL cells and N-terminal 10xHis-tagged recombinant RTA was purified using Ni-NTA agarose from QIAGEN (Valencia, CA, USA). The protein showed a single band on SDS-PAGE by Coomassie Brilliant Blue R-250 staining (Fig. S5) and by immunoblot analysis and was active in *in vitro* binding assays^67^.

### Surface Plasmon Resonance (SPR)

The interaction between RTA and the human P1/P2 complexes was conducted using surface plasmon resonance (SPR) with a Biacore T200 (GE Healthcare, USA) as previously described, with some modifications^38, 67^. The His tagged RTA was captured on the Ni^2+^–nitrilotriacetic acid (NTA) sensor chip as the ligand and the human P1/P2 complexes were used as an analyte. Stalk dimers at 50 nM were passed over the immobilized RTA at 50 µL/min for 5 min and the dissociation was for 3 min. The running buffer contained 20 mM Tris-HCl pH 8.0, 150 mM NaCl, 10 mM MgOAc, 0.005% surfactant P20.

### Isothermal Titration Calorimetry (ITC)

An isothermal titration calorimeter (Microcal ITC-200, USA) was used to measure the enthalpy and entropy changes during the interaction between RTA and the human P1/P2 complexes. Titrations were carried out using a 70 μL autopipet at 1000 rpm stirring speed. The sample cell (approximately 250 μL) was loaded with *RTA* solution (30 µM), and the autopipet was filled with different P1-P2 protein complexes in 1:10 ratio to RTA. The obtained raw calorimetric data were analyzed using the MicroCal Origin 7.0 software. The purified stalk dimers and truncated variants were dialyzed against 50 mM Tris buffer, pH 7.5, 150 mM NaCl, 10 mM MgCl_2_, 2 mM mercaptoethanol, 0.5 mM PMSF overnight at 4 °C and centrifuged at 15000 *g* for 15 min. Protein concentrations were determined from the absorbance at 280 nm using an extinction coefficient for each protein complex. All measurements were conducted at 30 °C.

### Microscale Thermophoresis (MST)

Purified RTA was labeled according to the manufacturer’s instructions with a Monolith NT RED NHS NT647 labeling kit (NanoTemper Technologies) using red fluorescent dye NT-647 *N*-hydroxysuccinimide (amine-reactive). The fluorescent dye to protein ratios were determined using photometry at 650 and 280 nm. Labeling reagents were removed by buffer-exchange column chromatography into MST reaction buffer: 50 mM Tris-HCl pH 7.4, 150 mM NaCl, 10 mM MgCl_2_, 0.05% Tween-20. The RTA protein samples were titrated with a serial twofold dilution with 1 mg/mL of bovine albumin. The concentration of labeled proteins was adjusted to approximately 5–20 nM. Twofold dilution series of up to 16 unlabeled P1-P2 protein concentrations were prepared in a final volume of 20 μL for the MST measurements starting from 50 to 100 μM. Thermophoresis was measured using a Monolith NT.115 pico instrument (NanoTemper) at an ambient temperature of 25 °C and 5/30/5 sec. laser off/on/off times, respectively. The instrument parameters were adjusted to 90% LED power and 20% MST power. The data were analyzed using NanoTemper’s NT analysis software (version 1.5.41) using the “Thermophoresis and T Jump” resulting signal. The change in thermophoresis was expressed as the change in the normalized fluorescence (ΔFnorm), which is defined as F_hot_/F_cold_ (F-values correspond to average fluorescence values between the defined areas marked by the red and blue cursors, respectively). Titration of the non-fluorescent ligand resulted in a gradual change in thermophoresis, which was plotted as ΔFnorm to yield a binding curve, which was fitted to derive the binding constants.

### Bio-Layer Interferometry Spectroscopy (BLI)

The BLI experiments were performed using an Octet RED96 system (ForteBio, Pall, UK). The His-tagged RTA was immobilized on the Ni^2+^- nitrilotriacetic acid (NTA) sensor chips (ForteBio, Pall, U.K.) as the ligand and the human P1-P2 stalk complexes were used as the analyte. NTA biosensors were hydrated in equilibration buffer (20 mM Tris-HCl pH 7.4, 150 mM NaCl, 10 mM MgCl_2_) for 10 min at room temperature. Experiments were set up in 96-well tray format in 200 μL volume of reaction buffer (20 mM Tris-HCl pH 7.4, 150 mM NaCl, 10 mM MgCl_2_, 0.01% Tween-20). After a baseline step of 60 seconds, RTA was immobilized on the NTA biosensors at a concentration of 0.06 μg/μL for 100 seconds with rotation at 1000 rpm. A second baseline step of 60 seconds followed the immobilization to wash unbound RTA and to allow for signal stabilization. The 160 second association, followed by 600 second dissociation protocol was used. The pilot experiment was performed for P1-P2 and ΔuL10(P1-P2)_2_ stalk complexes to determine the optimal protein concentrations for the kinetic analysis. The association was monitored by transferring the ligand biosensors to wells containing the stalk protein complexes in a concentration of 0, 14, 28, 56, 112, 225, 450 and 900 nM. For each experiment, the non-specific binding was monitored using a reference biosensor subjected to each of the above steps in the absence the RTA loading step. The y-axes of all steps were aligned to each step’s baseline, and curve fitting was performed to analyze the data. Curve fitting and K_*D*_ value determination was calculated using both the association and dissociation steps and a 1:1 global fitting model. The experiments were performed at least three times and the final K_*D*_ values reported are the averages of the replicates. The fitted curves with the highest R2 value were used in the analysis. All data were normalized to baseline and subtracted from minimal non-specific binding.

### Circular Dichroism (CD) Spectroscopy

CD spectra were recorded with application of a Chirascan Plus Spectrometer (Applied Photophysics, U.K.). The purified stalk dimers and their truncated variants were dialyzed against 50 mM Tris-HCl pH 7.4, 150 mM NaCl, 10 mM MgCl_2_. The protein concentrations were determined from the absorbance at 280 nm using an extinction coefficient for each protein complex. Spectra were recorded in the range 200–250 nm, at 25 °C temperature, with a 1 nm resolution. The scan rate was 60 nm/min. Protein samples for CD were scanned three times and averaged. The averaged baseline spectra were then subtracted from the averaged sample spectra and converted to molar ellipticity. The recorded spectra were analyzed with application of the Grams/AI software from Thermo Scientific (USA).

### Mass Spectrometry

All complexes were analyzed using SYNAPT G2-Si High Definition Mass Spectrometer (Waters, Manchester, U.K.). For the analysis, all protein solutions were buffer exchanged into 200 mM ammonium acetate (pH 7.5) using Micro Bio-Spin chromatography columns (Bio-Rad). Aliquots (∼2 μL) were introduced into the mass spectrometer via nanoflow capillaries using the following conditions: capillary voltage 1.2 kV, sampling cone 120 V, source offset 20 V. The source temperature was set up for 25 °C. The collision voltage was adjusted for the optimal signal level. Maximum entropy (MaxEnt, Waters) deconvolution was applied to electrospray data to recalculate the gas phase existing masses.

## Electronic supplementary material

Supplementary Information
