# Supplementary material for: Human ribosomal P1-P2 heterodimer represents an optimal docking site for ricin A chain with a prominent role for P1 C-terminus
Source: Sci Rep. 2017 Jul 17;7:5608. doi: 10.1038/s41598-017-05675-5 (PMC5514047; doi:10.1038/s41598-017-05675-5)
Supplement: Supplementary file 1 — Supplementary Information [file 41598_2017_5675_MOESM1_ESM.pdf]

Supplementary Information

Human ribosomal P1-P2 heterodimer represents an optimal docking site for ricin A chain with a prominent role for P1 C-terminus

Przemysław Grela[1,2], Xiao-Ping Li[1], Patrycja Horbowicz[2], Monika Dźwierzyńska[2], Marek Tchórzewski[2*] and Nilgun E. Tumer[1*]

[1]Department of Plant Biology and Pathology, School of Environmental and Biological Sciences, Rutgers University, New Brunswick, New Jersey, 08901-8520 USA,

[2]Department of Molecular Biology, Maria Curie-Skłodowska University, Akademicka 19, 20-033 Lublin, Poland

Supplementary methods

Isothermal Titration Calorimetry (ITC). An isothermal titration calorimeter (Microcal ITC-200, USA) was used to measure the enthalpy and entropy changes during the interaction between RTA and the human P1/P2 complexes. Titrations were carried out using a 70 μL autopipet at 1000 rpm stirring speed. The sample cell (250 μL) was loaded with RTA solution, and the autopipet was filled with different P1-P2 protein complexes solution (1:10). The obtained raw calorimetric data were analyzed using the MicroCal Origin 7.0 software provided with the instrument. Purified dimers and truncated variants were

dialyzed against 50 mM Tris buffer, pH 7.5, 150 mM NaCl, 10 mM $MgCl_2$, 2 mM mercaptoethanol 0.5 mM PMSF overnight at 4ºC. The solutions were then centrifuged 15000*g* for 15 min. Protein concentrations were determined from the absorbance at 280 nm using an extinction coefficient for each protein complex. All measurements were conducted at 30°C.

Supplementary figures:

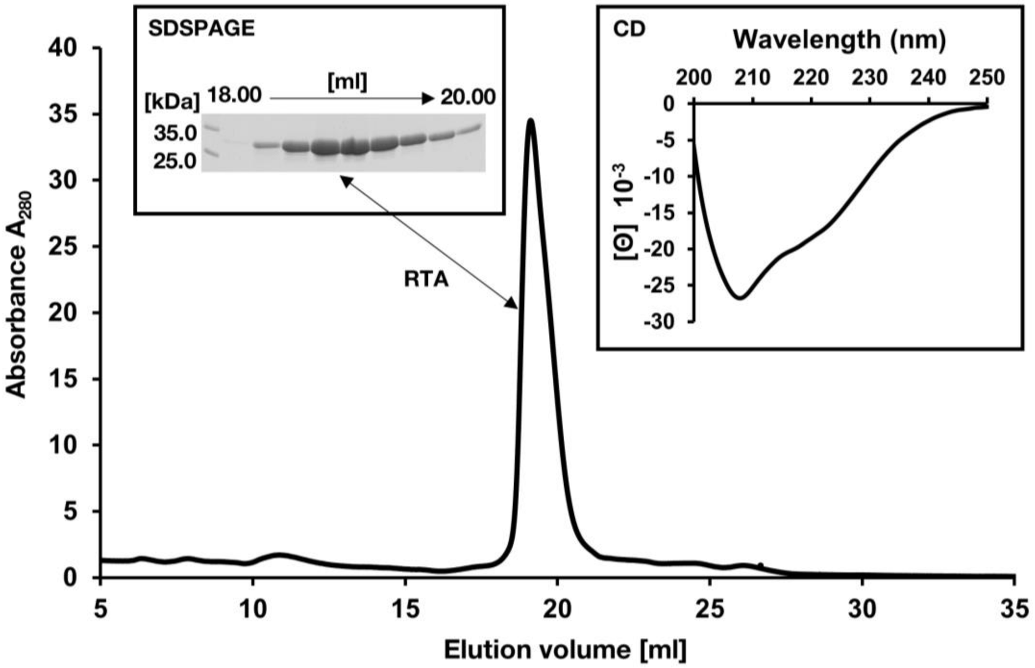

Supplementary Figure S1. Characterization of ricin toxin A chain (RTA). Purified RTA fractions were analyzed by size exclusion chromatography (SEC), SDSPAGE (left inset) and circular dichroism (CD) (right inset).

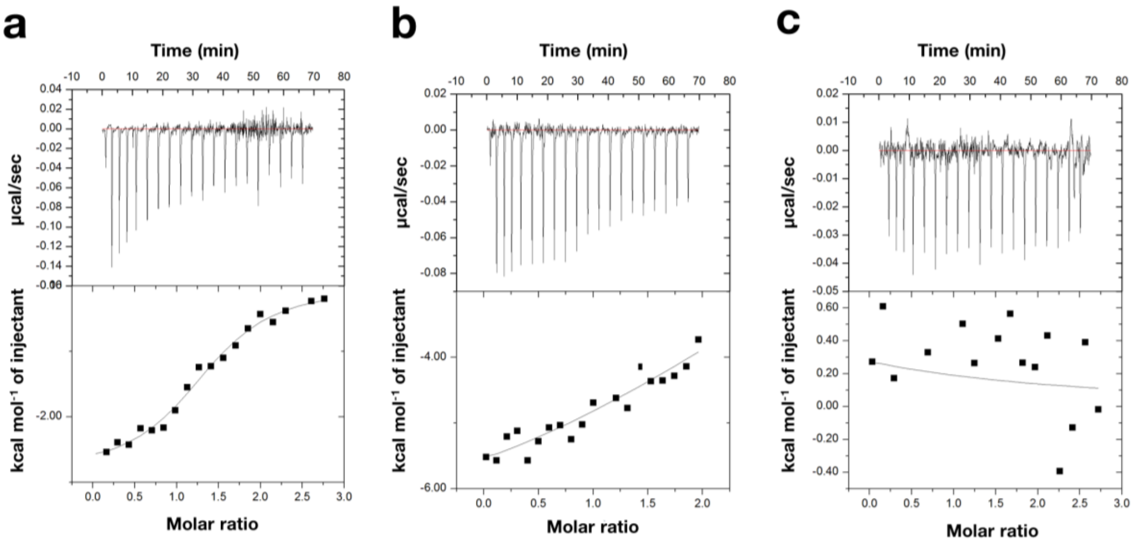

Supplementary Figure S2. Isothermal Titration Calorimetry (ITC) analysis of the interaction of RTA with the human ribosomal P1/P2 complexes. (a) Interaction of RTA with human P1-P2. **(b)** Interaction of RTA with human P1-P2ΔC. **(c)** Interaction of RTA with human P1ΔC-P2. The interactions were measured using a Microcal ITC-200. Buffer conditions for both RTA and human P1/P2 complexes consisted of 20 mM Tris pH 7.6, 150 mM NaCl, 10 mM $MgCl_2$, 2 mM mercaptoethanol, 1 mM PMSF. The differential heating power (Δ*p*) changes upon injection P1-P2 complexes into RTA solution (top). The integrated and normalized heat of reaction plotted against P1-P2/RTA molar ratio (bottom). The binding isotherms were fitted with a 1:1 binding model.

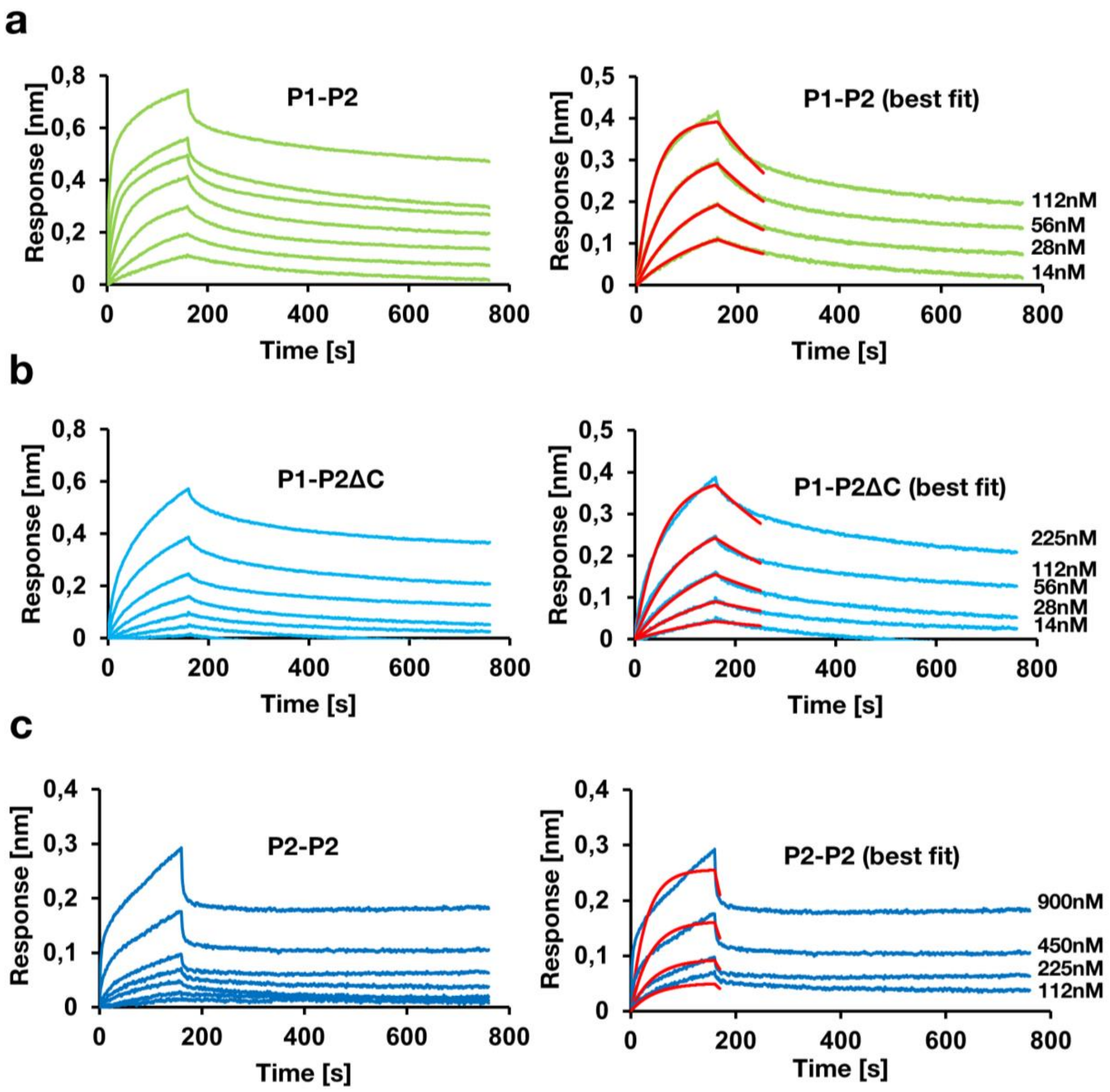

Supplementary Figure S3. Interaction of RTA with the human ribosomal P1-P2 dimers measured by BLI. (a) Interaction of RTA with human ribosomal stalk P1-P2 complex. **(b)** Interaction of RTA with human ribosomal stalk P1-P2ΔC complex. **(c)** Interaction of RTA with human ribosomal stalk P2-P2 complex. The left panels show binding curves for a concentration series of P1-P2 analyte at 0, 14, 28, 56, 112, 225, 450 and 900 nM. The right panels show representative fitted curves with the highest R^2 value. The sensorgrams are indicated by green lines and the corresponding fits to the 1:1 model by red lines. The data represent the mean values from three independent experiments.

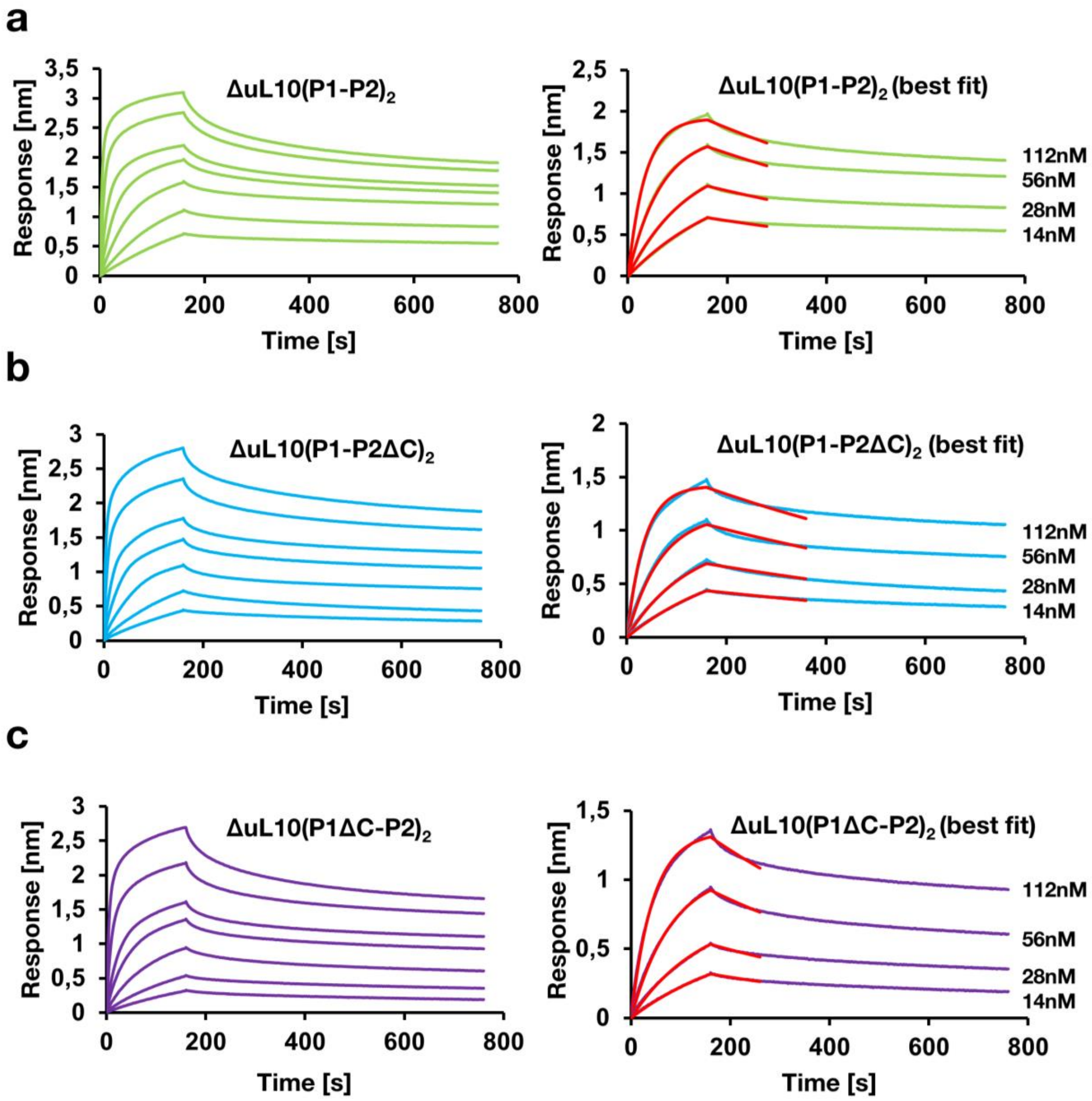

Supplementary Figure S4. Interaction of RTA with the different forms of human ribosomal stalk pentamer measured by BLI. (a) Interaction of RTA with human ribosomal stalk ΔuL10(P1-P2)$_2$ complex. **(b)** Interaction of RTA with human ribosomal stalk ΔuL10(P1-P2ΔC)$_2$ complex. **(c)** Interaction of RTA with human ribosomal stalk ΔuL10(P1ΔC -P2)$_2$ complex. The left panels show binding curves for a concentration series of human stalk analyte at 0, 14, 28, 56, 112, 225, 450 and 900 nM. The right panels show representative fitted curves with the highest R^2 value. The sensorgrams are indicated by green lines

and the corresponding fits to the 1:1 model by red lines. The data represent the mean values from three independent experiments.

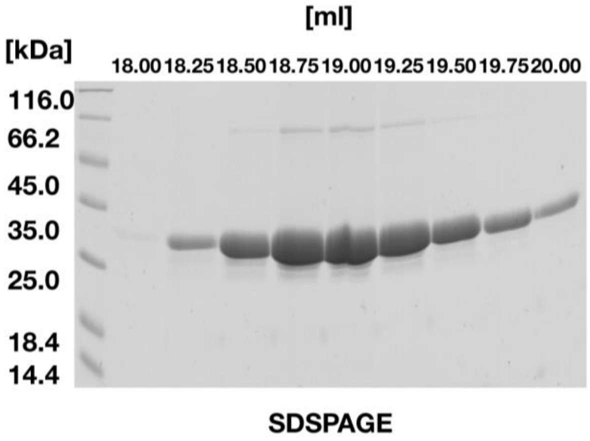

Supplementary Figure S5. SDSPAGE analysis of purified RTA fractions after SEC analysis shown in Figure S1. Twenty μL of each SEC fraction was analyzed by SDS-PAGE.

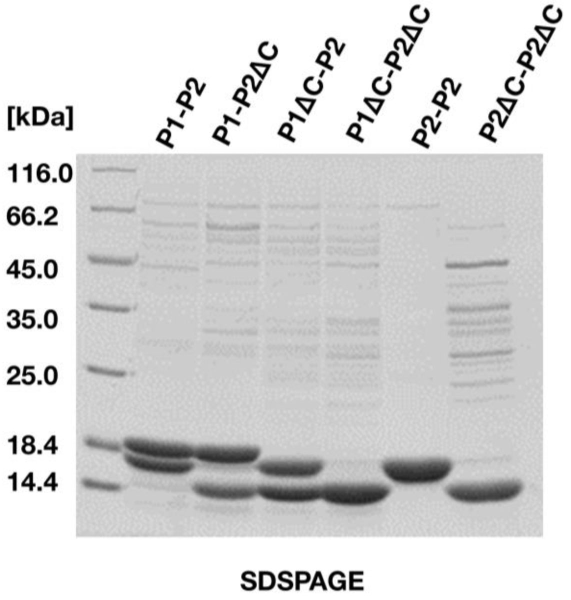

Supplementary Figure S6. SDSPAGE analysis of purified P1-P2 complexes shown in Figure 1. Ten μg of purified protein was analyzed by SDS-PAGE.

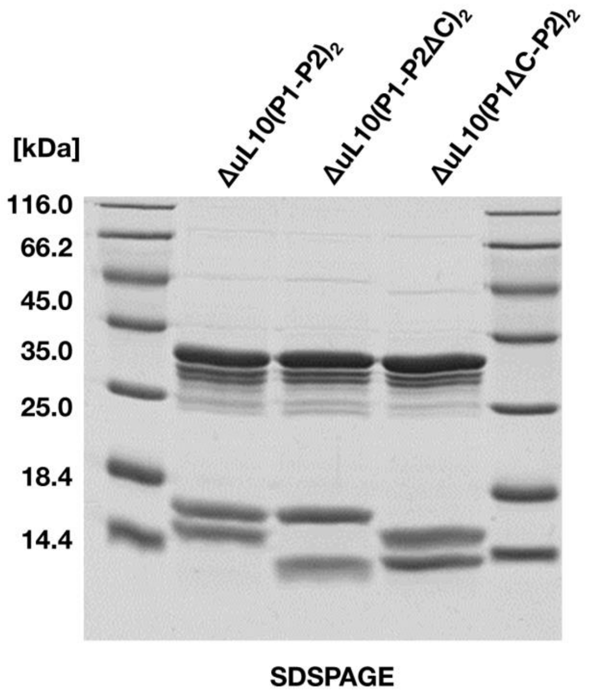

Supplementary Figure S7. SDSPAGE analysis of purified human stalk pentamer complexes shown in Figure 3. Ten μg of purified protein was analyzed by SDS-PAGE.
